# The effect of animated consent material on participants’ willingness to enrol in a placebo-controlled surgical trial: a protocol for a randomised feasibility study

**DOI:** 10.1186/s40814-021-00782-7

**Published:** 2021-02-08

**Authors:** Elizabeth Nelson, Cade Shadbolt, Samantha Bunzli, Angela Cochrane, Peter Choong, Michelle Dowsey

**Affiliations:** 1 The University of Melbourne Department of Surgery, St. Vincent’s Hospital, Level 2 Clinical Sciences Building, 29 Regent Street, Fitzroy, Melbourne, VIC 3065 Australia; 2 Department of Orthopaedics, St. Vincent’s Hospital, Level 3 Daly Wing, 35 Victoria Parade, Fitzroy, Melbourne, 3065 VIC Australia

**Keywords:** Recruitment, Informed consent, Feasibility, Arthroscopic subacromial decompression, Orthopaedics, Placebo-controlled surgical trial, Sham surgery

## Abstract

**Background:**

Placebo-controlled surgical trials are recognised as the gold standard way to test the efficacy of a surgical procedure. Despite a rise in arthroscopic subacromial decompression (ASD) surgeries for the treatment of shoulder pain, only two placebo-controlled surgical trials have been conducted. These trials encountered significant recruitment challenges, threatening the external validity of findings. Difficulties with recruitment are common in clinical trials and likely to be amplified in placebo-controlled surgical trials. This mixed method feasibility trial aims to address the following questions: (i) Feasibility: What proportion of patients who have consented to undergo ASD report that they would be willing to enrol in a placebo-controlled trial for this procedure? (ii) Optimisation: Can patients’ willingness to enrol in, or understanding of, such a trial be improved by supplementing written consent materials with a brief visual animation that outlines the details of the trial? And (iii) exploration: What factors influence patients stated willingness to enrol in such a trial, and how do they believe the recruitment process could be improved?

**Methods:**

This study aims to recruit 80 patients on the waiting list for ASD. Participants will be randomised (1:1) to either view a brief video animation explaining the hypothetical placebo-controlled trial in addition to written information or to written information only. Participants in both groups will be required to state if they would be willing to opt-in to the hypothetical ASD trial after immediately being presented with the consent material and again 1 week after completion of the consent process. Patients in both groups will also be required to complete a measure of trial literacy. Twenty participants will be purposively sampled to take part in an embedded qualitative study exploring understanding of trial concepts and factors contributing to willingness to opt-in.

**Discussion:**

This feasibility study will provide evidence for optimising participant recruitment into a placebo-controlled trial of ASD by consenting patients using animated trial information in addition to written information. This pilot and feasibility data may also be relevant to placebo-controlled surgical trials more broadly, which are characterised by recruitment challenges.

**Trial registration:**

ANZCTR, ACTRN12620001132932, date October 30, 2020

**Supplementary Information:**

The online version contains supplementary material available at 10.1186/s40814-021-00782-7.

## Background

Placebo-controlled surgical trials are considered the gold standard for testing the efficacy of surgical procedures [1]. Although important disagreements about how best to define the concept of a surgical placebo have yet to be resolved [2], it is widely accepted that these control interventions should at the very least aim to mimic the standard surgery so patients are unaware of which arm of the trial they are enrolled in, and exclude any parts of the procedure that are thought to have a therapeutic effect [1, 3]. By comparing the outcomes of patients who have been randomised to receive a placebo procedure to those who have had the standard surgery, trialists can assess the effectiveness of the standard therapy over and above non-specific and placebo effects. In orthopaedics, placebo-controlled surgical trials have helped change the practice landscape. The most well-known example of such changes relates to knee arthroscopy. Four placebo-controlled surgical trials have shown that knee arthroscopic lavage and debridement offer no benefit over and above the placebo effect [4–7]. Clinical guidelines now recommend against this procedure, suggesting that billions of dollars previously spent on these procedures every year should be put to better use [8, 9].

In light of these findings, placebo-controlled trials of other arthroscopic procedures are now garnering increased attention. One area of recent interest has been arthroscopic subacromial decompression (ASD) surgery for the treatment of shoulder pain [10, 11]. The two placebo-controlled trials of ASD conducted to date faced significant delays with recruitment. Comparing data from their clinical trial registry entries with their published results demonstrates that both trials ultimately required substantially more time to recruit than they had initially scheduled for both recruitment and patient follow-up. In both of these trials, the actual time to achieve target recruitment exceeded the target trial duration, with both trials requiring more than double the number of additional recruitment sites to achieve this. Difficulties with recruitment is common in clinical trials, with only one third meeting their target sample size [12]. This not only represents a significant waste of funds; it also raises ethical concerns. Patients who have enrolled in an underpowered or abandoned trial may have been exposed to unnecessary risk without contributing to the generation of new and meaningful medical knowledge [13]. The problem of low recruitment is likely to be amplified in placebo-controlled surgical trials. Patients may be reluctant to undergo an invasive placebo procedure without the expectation of some form of therapeutic benefit. Difficulties understanding concepts such as equipoise, placebo effects, randomisation, and blinding may also leave many patients confused and unwilling to participate in a placebo-controlled surgical trial [14].

Amongst the most commonly reported reasons for poor recruitment to surgical RCTs include difficulty understanding and a negative attitude towards trial concepts, and a preference for one form of treatment [14]. In line with these findings, Isaksson et al. [15] identified the two most important factors for enhancing recruitment in a RCT as (1) the research question needs to be considered important and (2) a simple procedure for providing information and gaining consent. One such strategy identified to help improve informed consent and therefore enhance recruitment includes the use of video animation. There is some evidence of the positive impact of video animation on enhancing patients’ understandability of research and attitude about participation in clinical research [16–19]. While two studies have shown that the use of educational videos do not, on their own, have a substantial impact on patients’ willingness to enrol in cancer-related trials [16, 17], the use of animated consent materials for placebo-controlled surgical trials has not been explored.

The challenges faced by previous trials highlight the pressing need to carefully evaluate the feasibility of placebo-controlled surgical trials early in the trial planning process. During the process of establishing the feasibility of such trials, there is also an imperative to assess the impact of concrete interventions that aim to optimise the trial’s likelihood of success. Moreover, there is a need to explore new avenues to optimise the feasibility of planned trials by engaging with key stakeholders. Taken together, these considerations provide a framework for this mixed-methods feasibility study, which aims to address the following questions.

- *Feasibility*. What proportion of patients who have consented to undergo ASD report that they would be willing to enrol in a placebo-controlled trial for this procedure?
- *Optimisation*. Can patients’ willingness to enrol in, or understanding of, such a trial be improved by supplementing written consent materials with a brief visual animation that outlines the details of the trial?
- *Exploration*. What factors influence patients stated willingness to enrol in such a trial, and how do they believe the recruitment process could be improved?

## Methods/design

### Trial design

To answer each of the research question outlined above, we will undertake a two-arm, randomised feasibility trial with an embedded qualitative component. The quantitative component of this study has been designed to test the hypothesis that supplementing written consent materials with a brief animated video leads to patients being more likely to consent to a trial comparing a placebo-control to ASD. This study will be carried out at St. Vincent’s Public Hospital Melbourne (SVHM) in Australia and will involve the recruitment of participant from both SVHM and from associated SVHM surgeon’s private clinic lists. The study strategy is registered, constructed, and presented according to the recommendations of the SPIRIT [20] and CONSORT extension for pilot and feasibility trials guidelines [21] (see Additional File 1). Ethical approval has been obtained from St. Vincent’s Hospital Melbourne Human Research Ethics Committee (reference number LRR 069/20, date 23 September, 2020). The trial has been registered with Australian New Zealand Clinical Trials Registry (ACTRN12620001132932, date October 30, 2020).

### Participants

Given orthopaedic surgeons in Australia often work in both public and private settings, participants will be identified from the SVHM orthopaedic public clinic lists and from the private rooms of surgeons who conduct surgery at SVHM. Individuals will be consented to participate in the feasibility study via telephone call with a member of the research team. After approximately 1 week, individuals will be contacted again to confirm participation. Patient-specific health and sociodemographic information will be collected to test for relevant differences between patients in each arm of the trial. A set of three questions will be used to assess functional health literacy (adapted from Ghanouni et al. [22]). These questions will specifically measure participants stated ease of understanding of medical statistics and written information, and an objective numeracy measure. In addition, we will measure trial literacy using the Therapeutic Misconception Scale. A measure of the participants’ general health status, including both physical and mental health, will be derived using the VR12 [23]. Symptom severity will be derived from a validated questionnaire (Oxford Shoulder Score [24]).

A subset of purposively sampled participants that represent a range of ages and gender from each study arm (approximately 20 in total) will be invited to participate in a phone audio-recorded qualitative interview. After the collection of all other outcome data, these participants will be asked to review the consent material (either written or video animation) while verbalising their immediate responses to these materials, using a ‘think aloud’ technique [25]. This will provide insight into how participants understand key concepts about the study design such as randomisation, blinding, and the use of placebo controls [26].

### Inclusion criteria

Patients will be considered for inclusion if they are the following:

- Are aged 18 years or older
- Have consented for arthroscopic subacromial decompression (ASD) with one of the orthopaedic surgeons from St. Vincent’s Hospital Melbourne (SVHM) or have consented to surgery at the private rooms of a surgeon who conducts surgery at SVHM
- Understand written and audio-visual instructions in English

### Exclusion criteria

Patients will be excluded from consideration if they are the following:

- Undergoing revision surgery
- Unable to provide informed consent due to mental incompetence (e.g., intellectual disability, dementia)
- Non-English-speaking

### Planned interventions

Participants will be presented with the informed consent materials for the arm to which they are randomised. Block randomisation will be used to allocate even numbers of participants to each arm. The consent materials used during the intervention will be presented to each participant individually, by one of the research team members.

#### Control

This group will receive a written participant information and consent form based on a hypothetical trial, using the template for interventional studies mandated by the Victorian State Government. The content of the consent form has been informed by Australia’s National Statement on Ethical Conduct in Human Research (NHMRC 2007) (see Additional File 2).

#### Intervention

In addition to the written participant information and consent form, participants randomised to the intervention arm will be provided with a 6-min-long video animation that explains a hypothetical trial in plain English. Content for the hypothetical trial is based on previously published placebo-controlled trials of ASD and informed by qualitative interviews with orthopaedic patients about their understanding of and attitude towards placebo-controlled surgical trials. The video animation will invite participants to take part in a hypothetical placebo-controlled trial of ASD. It will describe the procedure, the need for further research, what a placebo-controlled surgical trial involves, and trial participation requirements. It will also describe key concepts such as equipoise, placebo effects, randomisation, and blinding. All information represented in the consent video will also be contained in the written form.

### Outcomes

#### Primary outcome

The primary outcome is the proportion of participants who state that they would be willing to opt into the hypothetical ASD trial after being presented with the relevant consent materials. As is common with complex trials, participants will be encouraged to review and discuss trial information before, during, and after consenting to participate. Therefore, outcome measures will be collected immediately after participating in the informed consent process (*t*_2_) and at a second time-point 1 week after completion of the consent process (*t*_3_).

#### Secondary outcome

The secondary outcome, trial literacy, will be assessed using the Therapeutic Misconception Scale [27]. Therapeutic misconception occurs when research participants fail to distinguish between the imperatives of clinical research and ordinary treatment. Therapeutic misconception is detrimental to a participant’s understanding of a study, which is crucial for autonomous decision-making [27].

### Sample size justification

As a feasibility study, no formal power calculation is needed [28]. Based on recommendations for sample sizes for feasibility studies [29], we have set a maximum target of 40 participants per arm (approximately 80 in total) to ensure adequate representation of males and females from both public and private hospital settings. In qualitative research, data collection and data analysis are conducted in parallel and the sample size is determined by thematic saturation. As such, an a priori sample size calculation is not appropriate as it is not possible to know in advance when saturation will be reached. Previous qualitative studies embedded in orthopaedic trials have reached saturation at 12 [30] to 18 participants [31]. Therefore, the sample size of 80 participants will also be sufficient to enable the embedded qualitative evaluation.

### Randomisation and masking

To minimise bias, participants will be informed that the study aim is to explore patient understanding of informed consent procedures for a placebo surgery trial, but specific details about differences between these consent procedures will not be provided. Participants will only receive information on the mock trial arm they are randomised to and will receive no information about the comparator arm for the duration of the study. After being consented to this feasibility trial, participants will be randomly assigned in a ratio of 1:1 to either arm of the trial. Block randomisation will be performed by an investigator not involved in participant recruitment using computer-generated random assignment sequence prepared in blocks of four and stored in a password-protected file. A research assistant independent of recruitment and data collection will be responsible for participant management. The research associate (who will be responsible for patient consent) will be blinded to group allocation at the time of consent. In addition, outcome ascertainment will be blinded. Upon completion of the study, a biostatistician blinded to group allocation will analyse outcome data.

### Timelines

The anticipated start date of November 2020 and end date of December 2021. A summary for SPIRIT schedule of study including enrolment, intervention, and assessment is shown in Table 1.

- Patients will receive a participant information form (via email) and given a verbal explanation of the project via telephone. Patients will be consented to be a part of this trial using a verbal consent script, which will be audio-recorded and documented (see Additional file 2).
- At this point, patient-specific health and sociodemographic information will be collected.
- Once participants have agreed to be a part of the study, they will be randomly assigned to view one set of the consent materials for the hypothetical placebo-controlled ASD surgery trial.
- The main outcome measure will be participants stated willingness to opt-in to the hypothetical placebo-controlled shoulder surgery trial.
- Participants will also be asked a series of questions about their understanding of the information presented.
- This information will be collected at two time-points: immediately after reviewing and discussing the trial information with a researcher, and again approximately 1 week after completion of the consent process: 2 × 30-min sessions.
- After the collection of outcome measures, a purposively sampled subset of participants will participate in qualitative interviews.

**Table 1:** SPIRIT Trial study schedule of enrolment, interventions, and assessments

Time-point	Enrolment	Randomisation	Post-allocation			
	***− t***_**1**_	0	***t***_**1**_	***t***_**2**_	***t***_**3**_	***t***_***4***_
**Enrolment**						
**Baseline data, demographics**	X					
**Eligibility screen**	X					
**Informed consent**	X					
**Allocation**		X				
**Participant characteristics**						
**Health literacy**	X					
**General Health**^**a**^	X					
**Symptom Severity**^**b**^	X					
**Interventions**						
**Video animation**			X			
**Written information**			X			
**Assessments**						
**Recruitment rate**				X	X	
**Therapeutic misconception**^**c**^				X	X	
**Qualitative study**						
**Think aloud interview**						X

^a^VR-12

^b^Oxford Shoulder Score

^c^Therapeutic Misconception Scale

### Data management

All data will be stored on a password-protected computer kept in a secure locked facility and only accessible to the research investigators and the trial coordinator as approved by the SVHM Human Research Ethics Committee (HREC). At the completion of the study, outcome data will be pooled and de-identified for analysis by a statistician. Due to the short duration and minimal risks of the trial, there will not be a data monitoring committee. However, the principal investigator will be responsible for overseeing the trial and ensuring data quality and completeness, including participant enrolment, consent eligibility and forms, allocation to study groups, data recording and timeliness of data collection. Furthermore, there will be no planned interim analyses and stopping guidelines.

### Analysis

#### Quantitative analysis

Categorical variables will be analysed using chi-square tests. For continuous variables, we will employ (parametric) *t* tests and (non-parametric) Mann-Whitney tests for symmetrically and asymmetrically distributed data, respectively. Analyses will be conducted on an intention-to-treat basis by a blinded statistician using Stata, version 14.0 (StataCorp, College Station, Texas).

#### Qualitative analysis

The qualitative interviews will gain further insight into how participants understand key study design concepts, and further the understanding of factors that contribute to participants’ willingness to opt-in to the hypothetical trial. The interviews will be audio-recorded and transcribed data will be analysed using inductive thematic analysis [32] to compare data from participants randomised to written versus video animation informed consent. Concurrent with data collection, in the first stage of qualitative data analysis, two researchers (SB and EN) will independently identify concepts relevant to the research question in the first five interview transcripts. The two researchers will meet to discuss a preliminary coding framework, which will continue to be refined and extended through analysis of subsequent interviews. When the researchers are confident that the coding framework captures all relevant interview responses and no new codes are identified in subsequent interviews, recruitment will cease. Data will be uploaded into a qualitative data management software (Nvivo data management package, version 12.0) and all transcripts will be coded using the refined framework. Codes will be grouped thematically for each of the two study arms and themes between each study arm will be compared and contrasted. Emerging interpretations will be challenged through discussion amongst the inter-disciplinary research team.

## Discussion

The proposed study will provide evidence that will inform the planning and design of future placebo-controlled surgical trials. Not only will it provide information about the proportion of patients that can be expected to enrol in such a trial, which is directly relevant to its overall feasibility, it will also provide insights into whether recruitment could be optimised through the use of supplemental animated consent materials. By embedding a qualitative investigation into this study, we hope to gain further insights into other barriers and anxieties faced by patients when considering participation in such a trial. This will provide important information for educating researchers involved in the recruitment process. Even in trials that are otherwise feasible, improving the patient recruitment process is likely to reduce both the time and funding needed to conduct the trial. Although these findings will be relevant to many forms of placebo-controlled surgical trials, they are likely to be most generalisable to trials of ASD.

More generally, this study will contribute to our understanding of whether the use of animated consent materials improves trial recruitment. While two studies have shown that the use of educational videos do not, on their own, have a substantial impact on patients’ willingness to enrol in cancer-related trials (Du et al. 2008, 2009), the use of animated consent materials for placebo-controlled surgical trials has not been explored. Moreover, the only available study to assess the impact of providing a brief video in addition to written information showed that such supplemental information significantly increased willingness to enrol in a trial involving pregnant women with pre-labour rupture of membranes (Weston, Hannah, and Downes 1997). Importantly, this trial does not simply hope to test whether such supplemental information is likely to improve recruitment, it will also assess the impact of such interventions on patients understanding of the trial to which they are consenting. Improved understanding of such trials allows patients to more appropriately express their autonomy, which is vital to the ethical conduct of clinical research.

There are potential limitations of the proposed study. Firstly, we will only include English-speaking participants. As this is a feasibility trial, an important first step is to validate the intervention in English before it is applied to a larger cohort and translated into other languages. Secondly, the authors acknowledge limitations associated with the hypothetical nature of the placebo-controlled surgical trial that participants are to consider joining [33]. This may result in an over-estimation of participants’ willingness to opt-in. Consequently, recruitment rates reported in this trial will likely present an upper limit on the expected recruitment for a bona fide clinical trial in this domain. Importantly, the randomisation process should ensure that individuals’ propensity to overstate their willingness to participate in the hypothetic trial is similar between groups. Consequently, the findings of this feasibility study will provide actionable evidence about the relative effect of offering patients animated trial information in addition to written information.

## Supplementary Information

**Additional file 1:.** CONSORT extension for Pilot and Feasibility Trials Checklist

**Additional file 2:.** Verbal consent script and participant information form

## Data Availability

Not applicable

## Abbreviations

ASD: Arthroscopic subacromial decompression
RCT: Randomised controlled trial
SVHM: St. Vincent’s Public Hospital Melbourne
ANZCTR: Australian New Zealand Clinical Trials Registry
HREC: Human Research Ethics Committee
