# Supplementary material for: The effect of animated consent material on participants’ willingness to enrol in a placebo-controlled surgical trial: a protocol for a randomised feasibility study
Source: Pilot Feasibility Stud. 2021 Feb 8;7:46. doi: 10.1186/s40814-021-00782-7 (PMC7869245; doi:10.1186/s40814-021-00782-7)
Supplement: Supplementary file 2 — Additional file 2:. Verbal consent script and participant information form [file 40814_2021_782_MOESM2_ESM.docx]

**Verbal Consent Script**

**Introduction**

- My name is (*researchers’ name*) from (*organisational site*) and I am calling regarding your participation in the study ‘Optimizing Informed Consent for a Placebo-Controlled Surgical Trial’.
- Today’s date is the (day) of (month) 2020. Firstly, I would like to confirm that you are happy for this consent process to be recorded? *(await confirmation from participant).*
- I am confirming that you have received a copy of the participant information and consent form for the study ‘Optimizing Informed Consent for Placebo-Controlled Surgical Trial’? *(await confirmation from participant).*
- The aim of this research project is to explore your understanding of informed consent procedures for a mock trial comparing arthroscopic surgery for shoulder impingement with a placebo surgery.

**Description of participation**

- If you decide to take part in the research study, your participation will involve undergoing the consent process for a hypothetical trial comparing ASD surgery to a placebo surgery. This will take approximately 20 minutes and will be carried out over the phone with a researcher at {Site}. Immediately after, you will be asked about your willingness to participate in the hypothetical trial. You will also be asked a series of questions about your understanding of the information presented. This information will also be measured again approximately 1-week after completion of the consent process over the phone. At the completion of this follow-up you may also be asked to provide feedback on the consenting process with a researcher at a time convenient to you. We expect these questions to take approximately 10 minutes to complete.
- We don’t expect any risks or disadvantages associated with your participation in this study. However, if you experience feelings of distress as a result of participation in this study you can let the research team know and they will provide you with assistance.

**Data storage and use**

- The information you provide will remain confidential, and your personal information will not be disclosed in any publication that may result from this study.
- All information you provide during this study will be stored in a secure location.

**Withdrawal from the research**

- Please keep in mind that your participation is completely voluntary, and your decision to participate or not will not affect your care at {site}.
- If you decide to take part and later change your mind, you are free to withdraw from the study at any stage and don’t have to give a reason. If consent is withdrawn, the study will not collect additional information from you. Information that has been collected up to this point will be retained in the study, unless you ask for it to be deleted. Your decision to withdraw from the study will not affect your relationship with {site}.

**Questions**

Do you have any questions in regards to the information that I have provided?

- If yes, answer any questions the participant may have
- If no, continue to collect consent.

**Consent**

Now that I have explained what your involvement in the research study requires, are you happy to provide your consent to participate in the study?

- If no, thank the participant for their time and end the consent process.
- If yes, ensure you record the time and date the verbal consent has been collected from the participant. Furthermore, you will need to ask the participant if:
  - They are happy to be audio recorded

→ Commence with data collection.


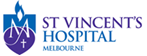


**Participant Information Sheet**

**Interventional Study** - *Adult providing own consent*

**St Vincent’s Hospital (Melbourne)**

| **Title** | Development and evaluation of informed consent procedures for recruitment into a placebo-controlled surgical trial |
| --- | --- |
| **Short Title** | Optimizing Informed Consent for Placebo-Controlled Surgical Trial |
| **Protocol Number** |  |
| **Project Sponsor** | The University of Melbourne, Department of Surgery, St Vincent’s Hospital, Melbourne |
| **Coordinating Principal Investigator** | Assoc/Prof Michelle Dowsey |
| **Location** | St Vincent’s Hospital (Melbourne) |

**Part 1 What does my participation involve?**

**1 Introduction**

You are invited to take part in this research project, ‘Development and evaluation of informed consent procedures for recruitment into a placebo-controlled surgical trial’. This is because you have consented to undergo surgery for shoulder impingement with one of the orthopaedic surgeons from St Vincent’s Hospital Melbourne. The aim of this research project is to explore patients’ understanding of informed consent procedures for a trial comparing arthroscopic surgery for shoulder impingement with a placebo surgery. In the study you have been invited to take part in, you will not be asked to take part in a placebo-controlled surgical trial. Instead, we will be exploring your attitude towards participating in a hypothetical trial and your understanding about the trial information.

This Participant Information Sheet tells you about the research project. It explains the research involved. Knowing what is involved will help you decide if you want to take part in the research. Please read this information carefully. Ask questions about anything that you don’t understand or want to know more about. Before deciding whether or not to take part, you might want to talk about it with a relative, friend or local doctor.

Participation in this research is voluntary. If you don’t wish to take part, you don’t have to. You will receive the best possible care whether or not you take part.

If you decide you want to take part in the research project, you will be asked to provide verbal consent over the phone with a researcher. By providing consent you are telling us that you:

• Understand what has been discussed with the researcher and what you have read

• Consent to take part in the research project

• Consent to the tests and research that are described

• Consent to the use of your personal and health information as described.

**2 What is the purpose of this research?**

Arthroscopic subacromial decompression (ASD) surgery for the treatment of shoulder pain is on the rise, with over 12,500 ASD currently performed in Australia each year. As with any surgical procedure, ASD is not without risk to patients, for example, the risks of a general anaesthetic and the risk of infection. To be sure that these benefits outweigh the risks it is important that researchers’ conduct clinical trials. This is where researchers test the surgical procedure against another procedure and see if the outcomes are different. The best way of doing this is to conduct a placebo-controlled trial. This is where a group of volunteers is recruited and half of them are given the real surgery. The other half undergo a pretend surgery made to look like the real surgery. To do this, the surgeon goes through the motion of surgery (i.e., giving anaesthetic, making a cut in the skin) but does not actually perform the procedure (i.e. no ligaments are cut and no tissue is removed from the joint). The researchers can see if the surgical procedure is effective or not by comparing the outcomes of volunteers who have had the real surgery with the outcomes of volunteers who think they have had the real surgery but in fact had the pretend surgery. If the outcomes of both groups are the same, then this tells the researchers that doing the real surgery (cutting ligaments, removing tissue in the joint) is not effective and exposes patients to unnecessary risks (e.g. infection). If the group who received the real surgery do better than the group who had the pretend surgery, then this tells the researchers that the surgery is effective.

Studies like these can change clinical practice. For example, placebo-controlled trials involving knees scopes to remove loose tissue in the knee thought to cause pain, have found that volunteers who receive a knee scope improve the same amount as volunteers who only receive a skin incision. This means that knee scopes are not as effective as we once thought, so clinical guidelines now recommend hospitals perform less of these procedures.

Not many placebo-controlled trials have been done for ASD, mostly because it is difficult to recruit volunteers who are willing to participate when there is a 50% chance they receive a real ASD, but also a 50% chance that they will have an anaesthetic, skin incision and nothing else done to their shoulder. We are conducting this study to understand if and when people undergoing ASD might be willing to participate in a placebo-controlled surgical trial. Your feedback is important to us because this will help us design future placebo-controlled surgical trials.

This research has been funded and supported through a seed grant, awarded to the University of Melbourne, Department of Surgery, St. Vincent’s Hospital.

**3 What does participation in this research involve?**

Participation in this research will occur during the normal process of preparing you for your ASD surgery. You will be invited to participate in this study by the study coordinator over the phone.

We will explain the study to you and you will have the opportunity to ask any questions you might have about the research. If you would like to participate in this study, we will ask you to provide verbal consent over the phone, which will be audio-recorded and documented.

If you agree to participate in this study, you will then undergo the consent process for a hypothetical trial comparing ASD surgery to a placebo surgery at a time convenient to you. The informed consent procedure for the hypothetical trial will take approximately 20 minutes and will be either via video-call or over the phone with a SVHM researcher. Immediately after you have reviewed and discussed the trial information with the researcher you will be asked about your willingness to participate in the hypothetical trial. You will also be asked a series of questions about your understanding of the information presented. This information will also be measured again approximately 1-week after the completion of the consent process over the phone or via video-call. At the completion of this follow-up patients may be asked to provide feedback on the consenting process with a researcher at a time convenient to you. We expect these questions to take approximately 10 minutes to complete. It is important to note that this will be recorded to help accurately capture your responses in your own words. A third party transcription service who adheres to the Commonwealth Privacy Act will transcribe your recording confidentially. Transcriptions will not be identified with your name or any other identifying information to reduce the risk of anyone knowing that you have participated in this study. Recordings will be kept in the strictest of confidence and not used for any other purpose, with all recordings destroyed following transcription.

In this study, there will be two different groups. A roughly even number of participants will be randomised into each group. The only difference between the groups will be the type of information you are presented with about the hypothetical placebo-controlled trial. Your planned ASD surgery will not be affected nor delayed by this participation in this study. You will continue to receive the care you would normally receive as a patient undergoing ASD surgery at St Vincent’s Hospital.

If you are a patient recruited from SVHM, information about your medical history and demographics including age, gender, medical conditions, socioeconomic data and cultural background, and x-rays will also be collected from your medical records.

There are no costs associated with participating in this research project, nor will you be paid to participate. You will be reimbursed for your time for participating in this study with a $25 gift voucher.

This research project has been designed to make sure the researchers interpret the results in a fair and appropriate way and avoids study doctors or participants jumping to conclusions. Members of the research team will meet on a monthly basis to discuss the progress of the study and ensure that the study is being conducted appropriately. Progress of the study will be reported to the Department of Surgery, St Vincent’s Hospital, the University of Melbourne.

**4 Other relevant information about the research project**

This study will be conducted at St Vincent’s Hospital Melbourne. It is anticipated that around 80 patients scheduled to undergo ASD will participate in this project, with approximately 40 patients in each group.

This project involves researchers from a number of organisations working in collaboration and includes researchers from The University of Melbourne, Department of Surgery, St Vincent’s Hospital, Melbourne.

**5 Do I have to take part in this research project?**

Participation in any research project is voluntary. If you do not wish to take part, you do not have to. If you decide to take part and later change your mind, you are free to withdraw from the project at any stage. Your decision whether to take part or not to take part, or to take part and then withdraw, will not affect your relationship with those treating you or your relationship with any of the institutions listed above.

**6 What are the possible benefits of taking part?**

There will be no clear benefit to you from your participation in this research. However, we hope that the results of this research will inform future research using placebo-controlled trials of ASD shoulder surgery.

**7 What are the possible risks and disadvantages of taking part?**

We do not anticipate that there will be any risks or disadvantages associated with your participation in this study, apart from the time to complete questions and the interview. It is not expected that there will be harm caused to you by being involved in this project. If you become upset or distressed as a result of your participation in the research, the study researcher will be able to arrange for counselling or other appropriate support. Any counselling or support will be provided by qualified staff who are not members of the research project team. This counselling will be provided free of charge.

**8 What will happen to my information?**

We will store the information we collect about you and your interview transcript on a secure, password protected computer file which will only be accessible by the researchers. We will keep this information for five years following publication of the research findings, which is consistent with the NHMRC Statement Guidelines on Research Practice. After this time, the data will be deleted.

**9 What if I withdraw from this research project?**

If you decide to withdraw from this research project, please notify a member of the research team before you withdraw. A member of the research team will inform you if there are any special requirements linked to withdrawing. If you do withdraw your consent during the research project, the study coordinator will not collect additional personal information from you, although personal information already collected will be retained to ensure that the results of the research project can be measured properly. You should be aware that data collected up to the time you withdraw will form part of the research project results. If you do not want them to do this, you must tell them before you join the research project.

**10 What happens when the research project ends?**

At the completion of this research project you will continue to receive the standard of care at St Vincent’s Public Hospital (Melbourne). The results of this study will be presented at conferences and submitted for publication in a peer-reviewed journal. It will also be presented to St Vincent’s Public Hospital (Melbourne).

**Part 2 How is the research project being conducted?**

**11 What will happen to information about me?**

By providing verbal consent you consent to relevant research staff collecting and using personal information about you for the research project. Any information obtained in connection with this research project that can identify you will remain confidential. We will remove any identifying information about you so that you cannot be individually identified. Your information will be stored in a secure password protected file; only the researchers will have access to this file. Your information will only be used for the purpose of this research project and it will only be disclosed with your permission, except as required by law.

Information about you may be obtained from your health records held at this health service for the purpose of this research. By consenting to this project you agree to the research team assessing health records if they are relevant to your participation in this research project.

It is anticipated that the results of this research project will be published and/or presented in a variety of forums. In any publication and/or presentation, information will be provided in such a way that you cannot be identified, thereby maintaining your privacy and confidentiality.

In accordance with relevant Australian and/or Victorian privacy and other relevant laws, you have the right to request access to your information collected and stored by the research team about you. You also have the right to request that any information with which you disagree be corrected. Please contact the research team member named at the end of this document if you would like to access your information.

Any information obtained for the purposes of this research project that can identify you will be treated as confidential and securely stored. It will be disclosed only with your permission, or as required by law.

**12 Who is organising and funding the research?**

This research project is being conducted and funded by the University of Melbourne, Department of Surgery, St. Vincent’s Hospital. No member of the research team will receive a personal financial benefit from your involvement in this research project (other than their ordinary wages).

**13 Who has reviewed the research project?**

All research in Australia involving humans is reviewed by an independent group of people called a Human Research Ethics Committee (HREC). The ethical aspects of this research project have been approved by the HREC of St Vincent’s Hospital, Melbourne. This project will be carried out according to the *National Statement on Ethical Conduct in Human Research (2007)*. This statement has been developed to protect the interests of people who agree to participate in human research studies.

**14 Further information and who to contact**

The person you may need to contact will depend on the nature of your query. If you want any further information concerning this project, you can contact the principal researcher, Assoc/Prof Michelle Dowsey on (03) 9231 3955 or the Study Coordinator Angela Cochrane on (03) 9231 2364 or Elizabeth Nelson on (03) 9231 3516. If you are undergoing surgery at SVHM and have any complaints about any aspect of the study or the way in which it is being conducted you may contact the Patient Liaison Officer at St Vincent’s Hospital Melbourne, with details provided below. You will need to tell the Patient Liaison Officer the name of the person who is noted above as principal investigator. Or, if you are undergoing surgery at the surgeon’s private rooms, please contact your surgeon’s private rooms. Regardless of where you are undergoing surgery, if you have any questions about your rights as a research participant, then you may contact the governance department (which is the approving HREC), which is provided below.

**Clinical Contact Person**

| Position | Patient Liaison Officer at St Vincent’s Hospital Melbourne |
| --- | --- |
| Telephone | (03) 9231 1954 |
| Email | [PLO@svhm.org.au](mailto:PLO@svhm.org.au) |

**Reviewing HREC Approving this Research** **and HREC Executive Officer Details**

| Reviewing HREC name | St Vincent’s Hospital Melbourne HREC |
| --- | --- |
| Position | HREC Executive Officer |
| Telephone | (03) 9231 2394 |
| Email | [research.ethics@svhm.org.au](mailto:research.ethics@svha.org.au) |
